# A Supervised Scene Adaptive Model for Identifying Impact Load with Few Samples

**DOI:** 10.3390/s25103169

**Published:** 2025-05-17

**Authors:** Shengbao Bai, Ji Yao, Chenhui Huang, Yuan Tian, Zhigang Xiong, Gang Chen, Hu Sun

**Affiliations:** 1 National Key Laboratory of Structural Integrity, Aircraft Strength Research Institute of China, Xi’an 710065, China; 2 National Key Laboratory of Ship Structural Safety, China Ship Scientific Research Center, Wuxi 214082, China; 3 School of Aerospace Engineering, Xiamen University, Xiamen 361005, China

**Keywords:** structural health monitoring, impact load detection, transfer learning, scene adaptive, deep learning

## Abstract

Deep learning-based impact load identification technology for the next generation of large aircraft structures has garnered significant attention and has become one of the focal points in aircraft structural health monitoring. However, this technology relies on a large number of training samples and exhibits poor scalability. One of the current challenges in system-level multi-structure monitoring is how to construct deep learning models with a small number or even zero impact training samples, and improve the models’ ability to migrate between different structures. To address this challenge, a novel method for impact load identification using only a small number of samples, based on a supervised scene adaptive model, is proposed. The performance of the model is validated on real aircraft structures. For large and complex structures, the model can be applied to other similar structural areas or different structural areas by using samples from the baseline area for training. Then, a very small number of calibration samples from the migrated area can be used for calibration. The results demonstrate that the proposed model, calibrated with just a single sample, achieves 97.22% accuracy in impact location identification and 99.44% accuracy in energy identification under similar regional structural conditions. Under different structural region conditions, the location identification accuracy of the proposed model is 87.65%, while the energy identification accuracy remains at 98.85%. The position identification accuracy of the model is 91.98% under different impact energy level conditions, and the identification accuracy remains at 87.04% even under varying impact energy levels and structural region conditions.

## 1. Introduction

Aircraft often experience numerous low-velocity impact events during flight. These impacts can result in easily detectable surface damage to the airframe, as well as barely visible damage within the structure itself. However, the internal damage caused by these impacts can pose a significant threat to the aircraft’s structural integrity and safety. If left undetected in real time, the expansion of such damage can prove fatal to the aircraft [1]. This issue is particularly pronounced in the new generation of aircraft that primarily use composites as their main material. Therefore, accurately identifying and assessing impact loads is crucial for ensuring the safe operation of an aircraft.

Low-velocity impacts generate dynamic stress waves that can be captured by piezoelectric sensors, allowing for the identification of impact location and load. The traditional method used for impact location identification is the time-of-arrival (TOA)-based method. This method determines the impact source location by analyzing the arrival time of the impact stress wave and establishing connections between wave velocity, time, and distance [2,3,4,5]. In isotropic structures, a system of equations can be constructed based on known sensor positions and acquired signals, enabling the recognition of impact positions when there are enough sensors to yield a system of equations with a rank greater than the number of variables [6,7,8]. However, for anisotropic structures, where stress wave propagation velocity varies with direction/angle, the set of equations becomes unsolvable due to added unknowns [9,10,11]. Consequently, the TOA-based method faces challenges when applied to complex structures with diverse material parameters, geometries, and boundary characteristics.

Another widely used method is the inverse problem method [12], which models signal characteristics from different impact locations to predict impact locations based on optimized models. However, this method struggles with determining the relationship between impact location and response signals, relying on variability magnitude between the signal to be identified and the model constructed with known signal characteristics.

A data-driven approach offers solutions to challenges encountered by traditional methods by training models using known impact response signals and establishing mapping relationships between impact response signals and impact positions [13,14,15,16,17,18]. This approach, encompassing system identification and machine learning methods, excels at modeling nonlinear relationships in complex structures without being limited by geometry or boundary conditions. The system identification method initially uses impact response signals from known locations to create a reference feature library. Subsequent impact response signals from unknown locations are then compared with the reference library, with the location corresponding to the most similar feature in the library being identified as the impact area [19,20]. However, when applied to larger structures, this method necessitates constructing a comprehensive feature library for the entire structure, demanding a considerable amount of data collection.

Machine learning methods, such as artificial neural networks (ANNs), back propagation networks, and convolutional neural networks (CNNs), have shown promise in fitting nonlinear relationships in complex structures. ANNs and back propagation networks require manually extracted features as input for impact location prediction. For instance, Seno et al. [21] utilized an artificial neural network model based on normalized smooth envelope (NSET) for impact localization on composite panels, achieving good results. Jang et al. [22] used fiber Bragg gratings for impact signal collection and location identification. Hao et al. [23] employed a back-propagation neural network for impact localization on anisotropic composite plates by extracting response signal arrival times as input features, yielding favorable recognition outcomes. Wen et al. [24] utilized fast Fourier transform and principal component analysis in a back propagation network model for better impact localization of CFRP laminates. Tabian et al. [25] introduced a novel model based on convolutional neural networks for load identification of composite panels, achieving high identification accuracy. Damm et al. [26] integrated polymer transfer molding, microelectromechanical systems, and piezoelectric sensors into carbon fiber-reinforced plastics, using deep learning for impact location and energy detection. Feng et al. [27] proposed an impact localization method based on time-frequency features of guided waves and convolutional neural networks, achieving improved edge location identification compared to arrival time-based algorithms.

Although machine learning methods offer improved fitting of nonlinear relationships in complex structures compared to traditional methods, they necessitate impact experiments across all scenarios, which can be impractical due to potential structural damage. Is there a method that can achieve superior identification by initially training with baseline scene samples and subsequently refining with a few transferred scene samples?

Transfer learning is a recognized solution to this challenge, widely applied in computer vision, fault diagnosis, and other fields. The typical strategy of transfer learning involves learning domain-invariant features from data, enabling models to maintain good predictive capabilities across different scenarios. Recent years have seen researchers propose several structural health monitoring methods based on transfer learning, achieving excellent performance in cross-domain structural health monitoring scenarios. Zhang et al. [28] introduced a multi-task integrated health monitoring method based on deep transfer learning, leveraging partial label signals for feature extraction model training with insufficient samples. Xu et al. [29] proposed a transfer learning-based method for predicting mechanical properties of composites, achieving similar accuracy with half the samples required by traditional CNN methods. Traditional transfer learning methods not only reduce the need for high-quality training samples but also enhance machine learning training efficiency. However, these methods still require a significant number of samples to fine-tune models in practical applications.

To address existing research challenges, this paper proposes an impact load identification method based on supervised scene adaptation, which combines deep learning’s automatic feature extraction capabilities with superior transfer performance and reduced sample requirements compared to traditional transfer learning methods. The method utilizes adversarial learning to find overlapping projections of impact response signals from baseline and migrated scenes in high-dimensional space, facilitating impact load identification with fewer samples under varying scene conditions.

The remainder of this paper proceeds as follows. Section 2 introduces the supervised scene adaptation-based impact load identification method with few samples. Section 2.1 analyzes the scene transfer problem of current impact load identification techniques, followed by Section 2.2 introducing the theoretical basis of the supervised scene adaptation method. Section 2.3 elaborates on the loss function and training strategy of the model, with Section 2.4 detailing the impact load identification process. Section 3 focuses on experimental validation, including dataset acquisition, processing, and model performance evaluation for impact location and energy identification in various scenarios. Finally, conclusions are drawn in Section 4.

## 2. Method and Process

### 2.1. Scene Transfer Problem Under Few Sample Conditions

Deep learning has achieved significant results in various applications, but they require large amounts of labeled training data. In addition, these techniques are developed based on the assumption that training and test data come from the same distribution. This constraint usually means that deep learning methods can only be specialized for specific structures and specific impact environments. However, in real engineering applications, the training and test data distributions are generally different. Subtle differences in the fabrication and installation of the components of two completely similar aircraft can also lead to differences in signal distribution. The signal distributions obtained from two completely similar structures, acquired in different operating environments, can also produce some differences. For different structures and unknown operating environments, the difference in the acquired signal distribution is even greater, and this phenomenon often occurs in actual operating scenarios. These issues can directly affect the generalizability of deep learning and thus lead to poor prediction performance. The current approach requires the collection of marker data for new scenarios and the simulation and massive data collection effort for each structure/area, working environment, and possible scenarios, which is certainly expensive and impractical. Collecting a large number of impact signals is bound to cause irreversible damage to the aircraft structure during the signal collection process, and requires a lot of manpower and resources to undertake the corresponding signal collection efforts. How to reduce the amount of data required by the model while maintaining the load identification capability of the model has become the focus of the current research on deep learning-based impact load identification methods.

In this paper, an impact load identification method based on supervised scene adaptation models is presented. The method uses a very small amount of target scene data to calibrate the model, and it can identify the impact response events of the target scene. The set of impact response signals acquired by the base scene is defined as the source domain, and the set of impact response signals acquired by the target scene is defined as the target domain. Here, it is assumed that the source and target domains have the same task labels and differ only in their respective data probability distributions. In order to obtain the desired load identification results, the proposed method should be able to learn domain-invariant features. If the features are domain-invariant, the model trained based on the baseline scene signal set should be able to recognize the signals of the target scene effectively. Therefore, learning domain-invariant features is a key process to accomplish impact monitoring.

**Figure 1:**
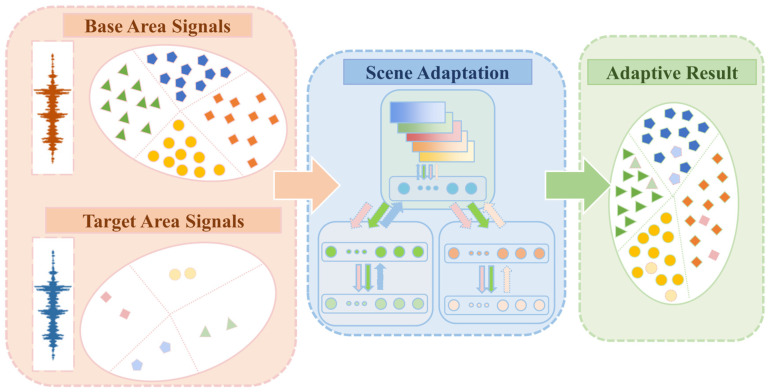
Principle and concept of supervised scene adaptation model.

The scene transferring based on the supervised scene adaptation method is illustrated in Figure 1, and the basic definition of scene adaptation is further explained in order to illustrate the problem to be solved more clearly. The scene adaptation problem is defined as follows, given a labeled baseline scene sample $D_{s}={\left\{x_{i},y_{i}\right\}}_{i=1}^{N_{s}}$ and a small number of labeled migrated scene samples $D_{t}={\left\{x_{j},y_{j}\right\}}_{j=1}^{N_{t}}$. The goal of scene adaptation is that the projections of the two scene datasets in both feature space and category space overlap in distribution $\gamma_{s}=\gamma_{t}$. When the joint probability distributions are different $P_{s}(x,y)\neq P_{t}(x,y)$, the baseline scene sample is used to learn the prediction function $f:x_{t}\to y_{t}$ on the migrated scene such that f has the minimum prediction error. The optimization objective is as in Equation (1):

(1) $$f=\arg\;\min_{(x,y)\in D_{t}}\varepsilon (f(x),y)$$

### 2.2. Supervised Scene Adaptation Model Framework

**Figure 2:**
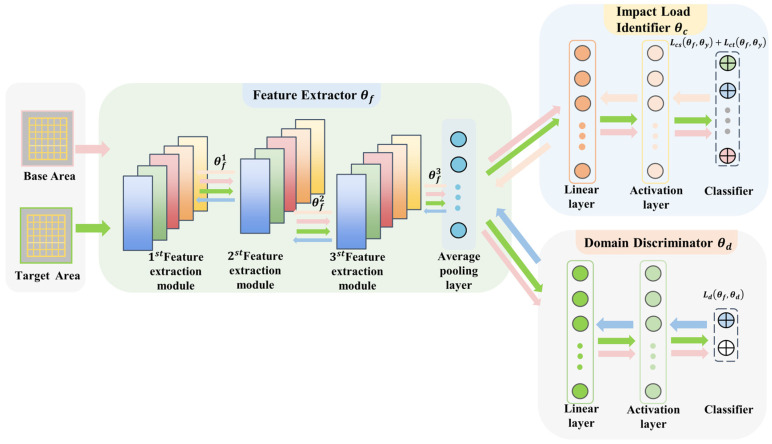
Structure of supervised scene adaptation model framework.

The model proposed in this paper is in the form of the scene adaptation model based on the idea of feature alignment. The model consists of three parts: feature extractor, load identifier and domain discriminator, and the structure of the model is shown in Figure 2. The feature extractor is used to extract domain-invariant features from the baseline scene samples and migrated scene samples, the load identifier is used to predict the impact location or energy level corresponding to the impact response signals from the baseline scene and migrated scene, and the discriminator is used to discriminate whether the input samples belong to the baseline scene or migrated scene. The discriminator is used to discriminate whether the input samples belong to the base scene or the target scene. The proposed model architecture is explained in detail in the following sections, and the selected components, the developed loss function and the training strategy are described in detail in the following sections.

#### 2.2.1. Feature Extractor

**Figure 3:**
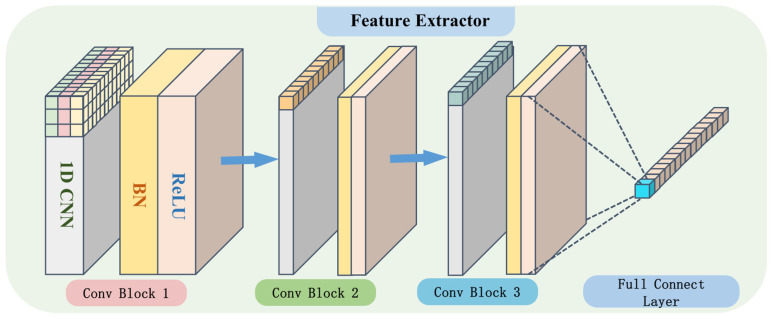
Internal structure of feature extractor.

The feature extractor mixes and maps the baseline scene samples and migrated scene samples to extract common spatial features. On the one hand, it makes the domain discriminator unable to distinguish which scene the data come from, and on the other hand, it extracts domain-invariant features, which makes the load identifier enough to identify the location or energy corresponding to the impact response signal. The feature extractor consists of three feature extraction modules, each of which is composed of one-dimensional convolutional neural network (1D-CNN), batch normalization (BN) and activation function ReLU. The structure of the feature extractor is shown in Figure 3.

The 1D-CNN fuses the fragment information of response signals in local receptive fields as well as the fused information of multichannel response signals to generate a new set of feature representations as outputs, and the relational equation of the feature representations is shown in Equation (2). In this way, the 1D-CNN performs convolutional operations on signal fragments of the input signal to extract the features associated with the corresponding impact locations, and then obtains the corresponding 1D feature mapping [30].

(2) $$x_{k}^{l}=\sum_{i=1}^{N_{l-1}}\mathrm{conv}(w_{ik}^{l-1},x_{i}^{l-1})+b_{k}^{l}$$

BN continuously adjusts the output after convolution so that the deep network can adapt to the parameter updates of the shallow network and the output of each layer tends to be stable, avoiding the disappearance of the gradient of the deep network leading to a decrease in the convergence rate, and also improving the generalization ability of the model through the correction of the data distribution during the learning process [31]. Equation (3) reveals the output after batch normalization $x_{i}^{l}$:

(3) $$x_{i}^{l}=\mathrm{BN}(x_{i}^{l})=\gamma (\frac{x_{i}^{l}-\mu }{\sqrt{\alpha^{2}+\varepsilon }})$$

Finally, the activation function is introduced to incorporate nonlinear factors to increase the model’s ability to fit the nonlinear system, thus solving the complex mapping relationship between the impact response signal and the impact load [32]. In this paper, ReLU is chosen as the activation function to avoid the gradient disappearance problem that may arise during the learning process, thus allowing the construction of a deeper model architecture [33]. The output $x_{i}^{l}$ after the nonlinearization of the activation function is as in Equation (4):

(4) xil=ReLU(xil)=xilxil>00xil≤0

In the proposed method in this paper, the sub-convolutional module does not use the conventional pooling operation. Since the local spatial features learned by the convolutional layer are subsequently used as inputs to learn the temporal dependence of the response signal in the time dimension, some important signal features may be lost if pooling measures are taken.

#### 2.2.2. Load Identifier

The role of the load identifier is to identify the samples from the baseline and transferring scenes and predict the corresponding impact location or energy as much as possible. The inputs to this module contain the feature information learned from the two scenes. The classifier consists of a linear layer, leaky ReLu activation function and output layer. The leaky ReLu activation function is a variant of the classical activation function ReLu, which has a small slope on the negative axis of the output. This setup causes the derivative to always be non-zero, thus reducing the appearance of silent neurons. The linear layer and leaky ReLu can be expressed as Equations (5) and (6), respectively:

(5) $$x_{k}^{l}=G(x_{i}^{l-1})=\sum_{i=1}^{N_{l-1}}w_{ik}^{l-1}x_{i}^{l-1}+b_{k}^{l}$$

(6) $$x_{i}^{l}=\mathrm{LeakyReLu}(x_{i}^{l})=\left\{\begin{array}{c} x_{i}^{l}\\ \alpha x_{i}^{l} \end{array}\right.\begin{array}{c} ,\\ , \end{array}\begin{array}{c} x_{i}^{l}>0\\ x_{i}^{l}\leq 0 \end{array}$$

The Softmax function is the activation function for multi-class classification problems, which is used in this paper for the prediction of impact regions. For any real vector of length K, the Softmax function can compress its value in the range [0,1] with length K and the sum of elements in the vector is 1. The specific formula is shown in Equation (7):

(7) $$p(y_{k}^{f_{c}}=j|x_{i}^{l})=\frac{e^{\left({\left(w_{ij}^{l}\right)}^{T}x_{i}^{l}+b^{f_{c}}\right)}}{\sum_{j=1}^{k}e^{\left({\left(w_{ij}^{l}\right)}^{T}x_{i}^{l}+b^{f_{c}}\right)}}$$

where {w,b} denotes the parameter between the linear layer and the output layer.

The loss function used by the load identifier is the cross entropy, which is shown in Equation (8):

(8) $$L_{c}^{i}=-\frac{1}{n_{l}}\left[\sum_{i=1}^{N_{l}}\sum_{j=1}^{k}I[y_{i}=k]\log\frac{e^{\left({\left(w_{j}^{l}\right)}^{T}x_{i}^{l}+b^{f_{c}}\right)}}{\sum_{j=1}^{k}e^{\left({\left(w_{j}^{l}\right)}^{T}x_{i}^{l}+b^{f_{c}}\right)}}\right]$$

where k is the number of delimited regions and I[⋅] denotes the indicator function. The optimal parameters of the load identifier are optimized by minimizing the loss function $L_{c}$.

#### 2.2.3. Domain Discriminator

The structure of the domain discriminator is similar to the load identifier, but the domain discriminator is used to determine the domain type of samples. Features extracted by the feature extractor $x_{i}^{l}$ are input to the domain discriminator. In the output layer of the domain discriminator, it is necessary to predict which domain the sample comes from, so the output layer is a binary classification prediction. Here, the sigmoid function is chosen to predict the domain labels, and the output result $d_{out}$ can be expressed as Equation (9):

(9) $$d_{out}=\frac{1}{1+e^{-({\left(w^{l}\right)}^{T}x_{i}^{l}+b^{f_{d}})}}$$

where {w,b} denotes the parameter between the linear layer and the output layer. When the output layer cannot accurately determine the domain class of the sample, the learned features can be considered as domain-invariant features. The loss function $L_{d}$ of the domain discriminator is as in Equation (10):

(10) $$L_{d}^{i}=t_{i}\log d_{out}(x_{i}^{l})+(1-t_{i})\log(1-d_{out}(x_{i}^{l}))$$

where $t_{i}$ denotes the domain labels 0 and 1, and $d_{out}(x_{i})$ is the predicted label. In the training process of the domain classifier, the loss function $L_{d}$ can be further derived as Equation (11):

(11) $$L_{d}^{i}=\frac{1}{N_{s}}\sum_{i=1}^{N_{s}}L_{d}^{i}(x_{i}^{l})+\frac{1}{N_{t}}\sum_{i=1}^{N_{t}}L_{d}^{i}(x_{i}^{l})$$

where $N_{s}$ and $N_{t}$ denote the number of samples in the baseline and target scenarios, respectively. The optimal parameters of the domain discriminator can be obtained by maximizing the loss function $L_{d}$.

### 2.3. Loss Function and Training Strategy

The supervised scene adaptation strategy employs the principle of feature distribution alignment, which maps the impact response signals in the source and target domains to a common feature space. The problem encountered by traditional methods is solved by aligning the feature distributions of the datasets in the source domain and the datasets in the target domain and making them indistinguishable. During the training process, the optimization objective of the model is to reduce the difference in distribution between the response signals in the source and target domains, and the difference in distribution between the response signals in the source and target domains is $d_{H}$, which is expressed as in Equation (12):

(12) $$d_{H}(L,\mathrm{UL})=\underset{\eta \in H}{\sup}\left|P_{x\sim L}\left[\eta (x)=1\right]-P_{x\sim \mathrm{UL}}\left[\eta (x)=1\right]\right|$$

$d_{H}$ represents the distribution difference between the source domain and the target domain, and is an indicator that measures the similarity of data distribution between the two domains. The smaller the value, the closer the data distribution between the source domain and the target domain. $\underset{\eta \in H}{\sup}$ means taking the maximum value in subsequent expressions. $P_{x\sim L}$ and $P_{x\sim \mathrm{UL}}$ represent the expected values of η with respect to x in the source and target domains, respectively. x is a randomly selected sample from the domain and calculates the average output value of η acting on these samples. The overall meaning of Formula (12) is to find the absolute value that maximizes the difference between the expected value of the source domain sample after being mapped by this function and the expected value of the target domain sample after being mapped by this function. This maximum value is the distribution difference measure between the source domain and the target domain. In the training process of supervised scene adaptive strategy, the optimization goal of the model is to reduce the distribution difference $d_{H}$, so that the response signals of the source domain and the target domain are distributed as similarly as possible in the feature space. This way, the model trained based on source domain data can be better applied to the target domain, achieving impact load recognition under few sample conditions.

The supervised scene adaptation strategy consists of three components: feature extractor $G_{f}$, load identifier $G_{y}$ and domain discriminator $G_{d}$. By introducing domain discriminators to determine whether the input samples come from the source or target domain, the features extracted by the feature extractor are also required to be able to deceive the domain discriminator, thus achieving a minimization of the distribution difference $d_{H}$ between the source and target domains. In the training period, on the one hand, the model tries to achieve the prediction goals that can be accomplished by traditional models and minimize the load identification error. On the other hand, the model tries to map the samples from the source and target domains to the common space through the feature alignment idea, both to minimize the difference between $G_{f}(x^{s})$ and $G_{f}(x^{t})$. The problem is a minimization–maximization problem in terms of the optimization of the loss function, which is as follows:

(13) $$L_{cs}^{i}(\theta_{f},\theta_{y})=L_{c}(G_{y}(G_{f}(x_{i}^{s};\theta_{f});\theta_{y}),y_{i}^{s})$$

(14) $$L_{ct}^{i}(\theta_{f},\theta_{y})=L_{c}(G_{y}(G_{f}(x_{i}^{t};\theta_{f});\theta_{y}),y_{i}^{t})$$

(15) $$L_{d}^{i}(\theta_{f},\theta_{d})=L_{d}(G_{d}(G_{f}(x_{i};\theta_{f});\theta_{d}),d_{i})$$

(16) $$L=(L_{cs}^{i}(\theta_{f},\theta_{y})+\mu L_{ct}^{i}(\theta_{f},\theta_{y}))-\lambda L_{d}^{i}(\theta_{f},\theta_{d})$$

where $x_{i}^{s}$ represents the *i*-th input sample in the source domain, $x_{i}^{t}$ represents the *i*-th input sample in the target domain. $\theta_{f}$ represents the features extracted using feature extractor $G_{f}$, $\theta_{y}$ represents the prediction result of load identifier $G_{y}$, and $\theta_{d}$ represents the output label of domain discriminator $G_{d}$. The loss function is divided into two parts: load label prediction loss (see Equations (13) and (14)) and scene label prediction loss (see Equation (15)), and the specific loss function is shown in Equation (16). The first term is the weighted sum of the load identification losses in the source and target domains, which aims to train the feature extractor and load identifier. The second term is the domain discriminant loss term for the source and target domains, which is to ensure that the signals from both domains are mapped to the common space. This minimization–maximization problem is solved by adding the gradient backpropagation layer between the feature layers, with the following backpropagation process, as shown in Equations (17)–(19):

(17) $$\theta_{f}\leftarrow \theta_{f}-\alpha (\frac{\delta L_{c}}{\delta \theta_{f}}+\lambda \frac{\delta L_{d}}{\delta \theta_{f}})$$

(18) $$\theta_{c}\leftarrow \theta_{c}-\alpha \frac{\delta L_{c}}{\delta \theta_{c}}$$

(19) $$\theta_{d}\leftarrow \theta_{d}-\alpha \frac{\delta L_{d}}{\delta \theta_{d}}$$

where α is the learning rate.

As the network is optimized to map the response signals from both domains into a common space, the load identifier can accurately identify unlabeled samples in the target domain.

Although the adversarial learning framework involves multiple network components (feature extractor, load recognizer, domain discriminator), its training complexity is increased compared to traditional single-task models [34]. However, the feasibility of deploying the model in practical engineering is guaranteed through the following design optimizations:

(1) Lightweight network structure: The feature extractor adopts a 1D-CNN module with only 10–20% of the parameter count of traditional 2D-CNN, and further compresses redundant parameters through pruning technology, making it suitable for deployment on embedded devices.

(2) Staged training strategy: Adopting a pre-training fine-tuning mode, the baseline scene model (source domain) can be trained offline, and only a small number of calibration samples are needed for fine-tuning when transferring to the target scene, greatly reducing the computational burden on site.

(3) Real time guarantee: The testing and verification time for a single impact signal is extremely short, meeting the real-time requirements for aircraft structural health monitoring.

### 2.4. Impact Load Identification Process

For the scene transferring and few-sample dilemma in impact load identification, this paper proposes a supervised scene adaptation model architecture. Based on the impact response signals of the base scene and the migrated scene, a series of signal processing is performed. Then, by aligning the features, the feature extractor has the ability to extract the features of different scene response signals at the same time, so as to achieve scene transferring and load identification with few samples.

**Figure 4:**
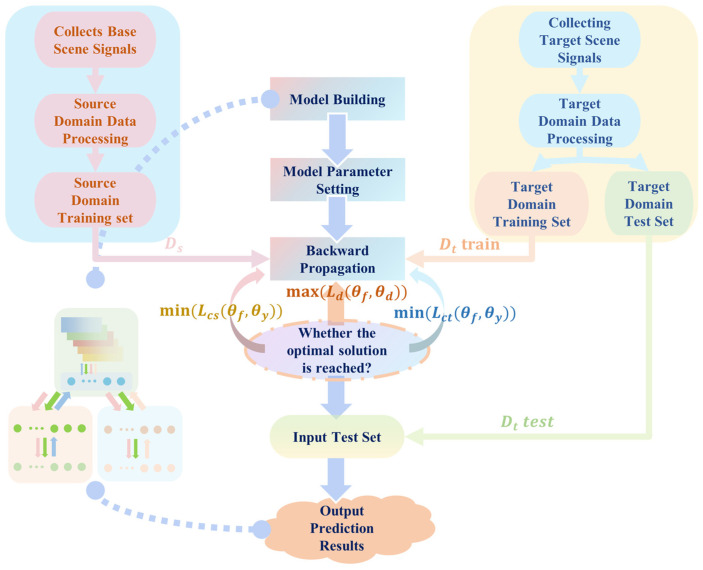
Flow chart of impact load identification with few samples based on supervised scene adaptation model.

The flow of the proposed method is presented in Figure 4. The process is summarized as follows:

Step 1: Label the impact response signal of the baseline scene and the impact response signal of the target scene with position/energy level labels and domain labels, and preprocess the response signal with the corresponding data.

Step 2: Build the supervised scene adaptation model, develop the training strategy of the model, and initialize the parameters and hyperparameters of the model.

Step 3: Perform model training and optimize the model parameters by back-propagation iteratively. Optimize the feature extractor and load identifier first, then optimize the domain discriminator, and repeat the above steps until the load identifier achieves a better identification effect.

Step 4: Test the model by using samples of transferred scenes as input to the model, and subsequently, the load identifier outputs the prediction results.

In this paper, Accuracy is introduced as an evaluation metric when performing impact load identification tasks. This evaluation metric can be expressed as follows:

(20) $$\mathrm{Accuracy}=\frac{m}{M}$$

where m is the number of correctly predicted samples and M is the total number of samples involved in the prediction.

## 3. Experimental Validation

### 3.1. Signal Acquisition and Processing

**Figure 5:**
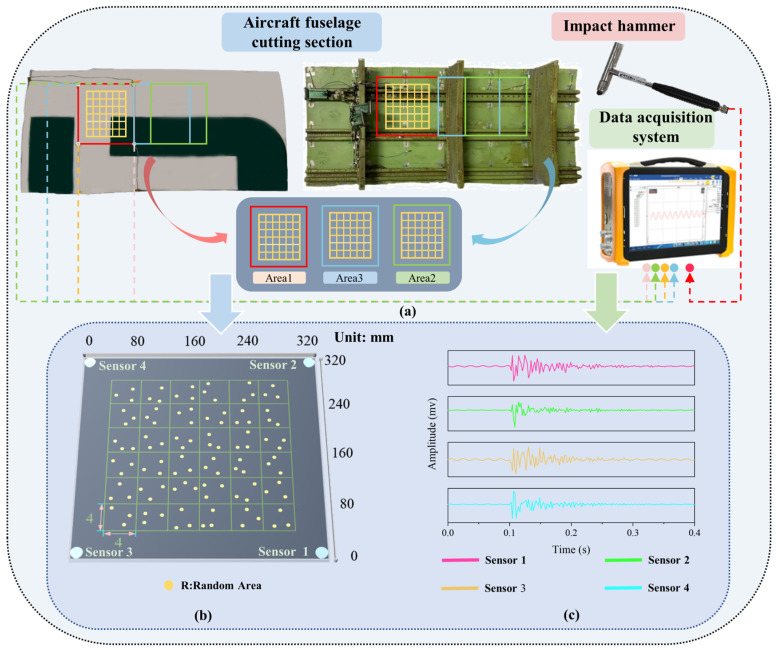
The layout of the impact experiment. (**a**) Experiment setup and arrangement; (**b**) area division and impact location calibration; (**c**) impact response signal.

In order to investigate whether the proposed model can achieve high accuracy of load identification under different impact conditions and different structural conditions, in this experiment, the structure cut section of the aircraft was selected as the research object, whose back contains longitudinal and transverse reinforcement structures with complex structural characteristics of real aircraft. An impact test platform was designed based on this fuselage structure cut section, as shown in Figure 5, to simulate the impact scenarios under different impact conditions and different structural conditions.

In order to validate that the model can complete the load identification transfer in different areas of the fuselage cutting section, three areas are selected for impact according to the structural characteristics: Area1, Area2 and Area3, where Area1 is the reference area, which has two reinforced structures crossing transversely and no reinforced structures crossing longitudinally and is 5 cm away from the nearest longitudinal reinforced structure. First, to validate whether the proposed model can accomplish better transfer in similar structural areas, Area2 is selected with similar structural conditions as Area1, which also has two reinforcement structures crossing in the transverse direction and is 5 cm away from the nearest longitudinal reinforcement structure. To further discuss the generalization ability and small sample transfer ability of the model, Area3 with completely different area structure conditions from Area1 and Area2 was selected, containing two horizontal structures and two vertical structures. The sizes of Area1, Area2 and Area3 are all 240 mm × 240 mm, and each local area is divided into 36 sub-areas, and each sub-area is a regular grid of 40 mm × 40 mm. Four PZT piezoelectric sensors were arranged around the three different areas to receive the impact response signals, and the arrangement and number of piezoelectric sensors are shown in Figure 5b. The sensors are PZT-5 type piezo-ceramic plates with Φ5 mm × 0.5 mm, the dielectric constant of this type of PZT is 2000 and the piezo-strain constant d_33_ is in the range of 420–620.

The impact is generated by the impact force hammer KSI-728A001 (Keshan Technology Co., Ltd., Beijing, China). The impact energy level of the impact force hammer is divided into low-energy impact and high-energy impact. The impact energy level of low-energy impact is below 200 N, while that of high-energy impact is above 200 N. In the practical impact process, instead of performing a fixed strength impact, random strength size impacts are performed within the delimited energy level range, so as to better verify the algorithm’s ability to identify arbitrary impact energy levels.

The impact response signals were acquired by the data acquisition device DH5925N (Donghua Testing Technology Co., Ltd., Taizhou City, China). For each impact, the sensor signals at each location were recorded at 50 KHz sampling frequency, and the number of acquisition points was 750,000. Continuous impacts containing two impact energy levels were performed in Area1 using the impactor, with each location containing 10 impacts. To further validate the algorithm’s ability to transfer areas and to transfer at different impact energy levels, a series of experiments were performed in Area2. The experiments were performed in Area2 using the impact head for two different impact energy levels, with five consecutive impacts for each case. To further validate the model’s ability to migrate in the area, impact experiments were conducted in Area3 for two different impact energy levels, with five consecutive impacts for each case. The 10/5 impacts do not occur at exactly the same location within each small square area. Our design allows for a certain amount of randomness in the impact points within the small square area in order to simulate the variety of impacts that may be encountered in actual use. The collected datasets are shown in Table 1. Since the method in this paper is based on 2D image input, the discrete signals from the sensors need to be processed into the appropriate format to facilitate subsequent processing. This format allows complete characterization of the signal, such as signal arrival time and signal amplitude, thus ensuring that this important information is fully retained.

### 3.2. Location Identification of Impact Load

In this section, the supervised scene adaptation model is used to predict the impact locations and to validate the transfer ability of the model under different structures and different impact energy levels. Here, the supervised scene adaptation model is compared with the original FCN model without transfer learning and the FCN network model with transfer learning to check whether the proposed model has improved generalization ability compared with the traditional deep learning model and the transfer learning model. Firstly, validate whether the model can transfer better under similar area structure and different area structure conditions, secondly, verify the generalization performance of the model under different impact energy level conditions, and finally, discuss the transfer effect of the model under mixed scenarios with different structures and different impact energy levels.

**(1) Transfer ability of model in similar area/structure scenarios**

First, the transfer ability of the model was verified in similar area/structure scenarios. For this research objective, two datasets were selected for training, which were from Area1 and Area2. High-energy impact A1B from Area1 was selected to form the baseline set. The same treatment was performed for the dataset from Area2, and the high-energy impact dataset A2B was selected to divide the dataset into a calibration set and test set accordingly.

**Figure 6:**
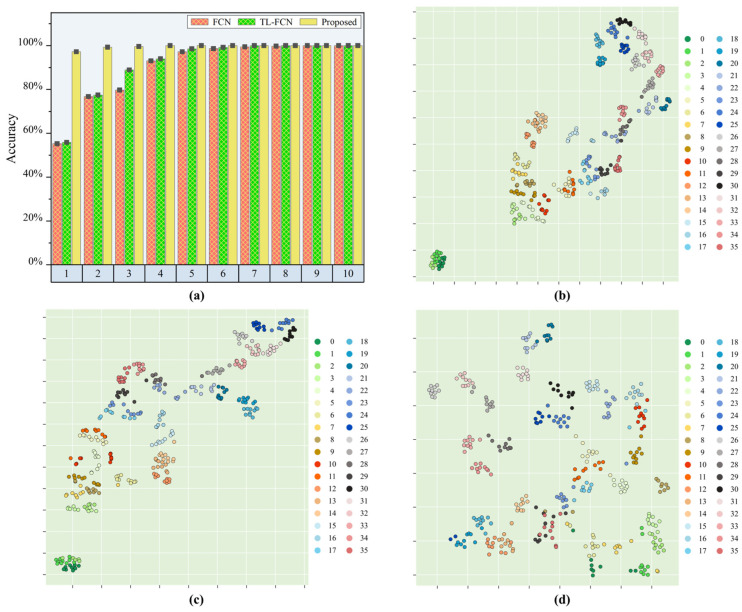
Load position identification effect of each model in similar area/structure scenarios. (**a**) Identification accuracy of each model; (**b**) feature visualization of the baseline FCN model; (**c**) feature visualization of TL-FCN model; (**d**) feature visualization of supervised scene adaptation model.

After experimental validation, the experimental results are presented in Table 2 and Figure 6. When there was one sample from each sub-region of Area2 involved in the calibration, the proposed model could achieve high accuracy recognition of Area2 with a prediction accuracy of 97.22%. The proposed model achieved significant improvement in load identification compared to the baseline FCN model, with an improvement of 41.97%, while the traditional transfer learning-based FCN model only improved by 0.58%. The proposed model achieved 99.3% accuracy in identifying samples from the target region Area2 when two samples from each sub-region of Area2 were involved in the calibration. The improvement was significant compared to the baseline FCN model, with 22.56% accuracy improvement. In contrast, the FCN model that performs transfer learning had only a small improvement in prediction effectiveness compared to the benchmark FCN model, with an improvement of only 0.69%. The prediction accuracy of the proposed model reached 100% when the calibration samples for each subregion were boosted to 4. From experiment validation, it was found that when the number of calibration samples in each sub-area reaches more than two, the identification ability of the proposed model for the target region remained basically unchanged even if the number of sub-area samples increased again. This was because the perfect projection of the impact response signals from the reference region and the target region to the common feature space could be completed with only two samples in the target region, so the improvement of the identification effect by increasing the calibration samples was not significant. When the calibration samples were less than 4, the identification effect of the proposed method was much better than that of the traditional transfer learning and benchmark model, especially when the calibration samples totaled 1, when the calibration effect was especially obvious. Therefore, it could be inferred that the proposed model had good engineering application value.

In order to understand more visually the transfer capability of the proposed model under similar structural conditions, the fully connected layer outputs of the baseline FCN model, the pre-trained TL-FCN model and the supervised scene adaptive model were further projected into the two-dimensional space using t-SNE. Figure 6b–d represent the feature distributions of the FCN model, TL-FCN model and supervised scene adaptation model for the test set predicted with the calibration set of one sample per sub-area. Ten samples were selected for tracking in each impact region, and the identification accuracy of each model was analyzed by identifying the distances within and between classes in the feature distribution plots. From Figure 6b, it can be seen that the baseline FCN model has a huge error in predicting the test data, and the distances of the impact response signal feature distributions in different sub-regions are small and almost mixed together. The small difference between Figure 6b,c indicates that the traditional transfer learning approach has very limited performance improvement in this case of very few samples. The feature distribution of the supervised scene adaptive model for prediction of test data is shown in Figure 6d, and it can be clearly seen that the impact response signal features in different sub-regions are distributed at a large interval, and the impact response signal features in the same sub-region also achieve effective clustering.

The above identification results and t-SNE feature analysis indicated that the proposed model could accomplish the transfer of similar structures well, and after a certain amount of baseline data, similar structures could be identified with high accuracy by the model with only a very small amount of calibration data collected from the baseline region and the target region. And compared with the traditional FCN model and TL-FCN model, there was a great improvement in the identification accuracy of few samples.

**(2) Transfer ability of model in completely different scenarios of area/structure**

This subsection continues to discuss the transfer capability of the model under completely different area/structure conditions, and further verifies whether the model could accomplish the transfer in any area/structure. To validate this goal, datasets A1B and A3B from Area1 and Area3, respectively, were selected. A1B was used as the baseline dataset for the initial training of the model, and A3B was divided into calibration and test sets to complete the calibration and testing of the model.

**Figure 7:**
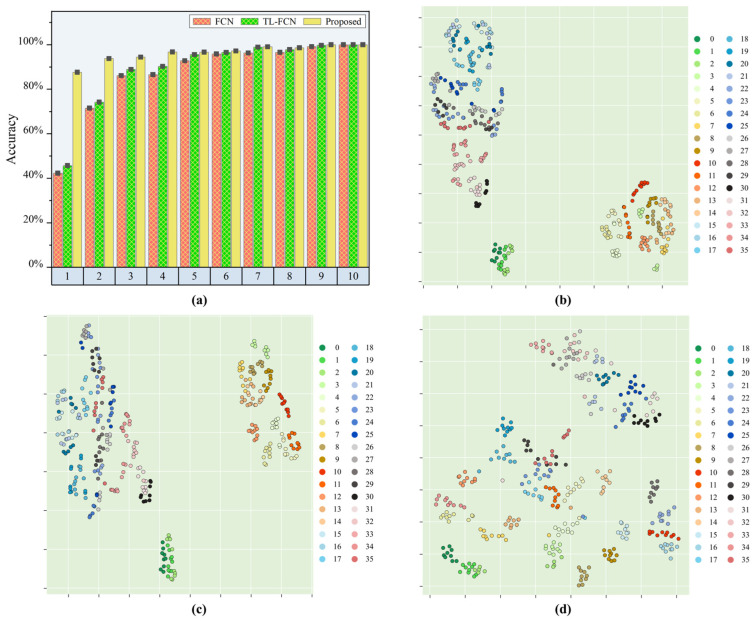
Load position identification effect of each model in different area/structure scenarios. (**a**) Identification accuracy of each model; (**b**) feature visualization of the baseline FCN model; (**c**) feature visualization of TL-FCN model; (**d**) feature visualization of supervised scene adaptation model.

The results shown in Table 3 and Figure 7 were obtained after experimental validation. The identification accuracy of the proposed method was twice as high as that of the baseline FCN model when the calibration sample for each subregion was one, with an improvement of 45.37%. In contrast, the optimization effect of the traditional transfer learning-based FCN model with one calibration sample was small, and the prediction accuracy was only improved by 3.4% compared to the benchmark FCN model. When the calibration sample was increased to 2 for each subregion, the proposed method achieved 93.75% load identification accuracy, which was 22.22% higher than the benchmark FCN model. In comparison, the improvement of the traditional transfer learning-based FCN model was still limited to 2.64%. The load identification capability of the proposed model increased with the increase in calibration samples, and the load identification accuracy reached over 95% for four calibration samples in a single sub-area and over 99% for seven calibration samples in a single sub-area. In comparison, the identification effect of the baseline FCN model and the traditional transfer learning-based FCN model with very few calibration samples was very limited, and the identification accuracy of a single sub-area with less than three calibration samples was less than 80%. It was also found that the load identification improvement effect of the FCN model with the introduction of traditional transfer learning mechanism was also very limited, and the improvement effect was less than 5%. Through further experiments, the proposed model still had high identification accuracy for the different target area structures from the baseline area structure, and only one calibration sample for a single sub-area was needed to calibrate the model to obtain a good result. Compared with the baseline FCN model and the traditional FCN model based on transfer learning, the proposed model showed excellent performance in different area structure scenarios and had good engineering applicability.

The t-SNE could observe the effect of transfer learning strategy and scene adaptation strategy more intuitively. Therefore, the fully connected layer high-dimensional output vectors of the baseline FCN model, the pre-trained TL-FCN model and the supervised scene adaptation model were transformed into two-dimensional features by t-SNE here, and the respective feature distributions are plotted in Figure 7b–d. Here, the feature distributions of each model after feature extraction for a test set with a single sub-area of ten samples were analyzed when the calibration dataset was a single subregion of one sample. Figure 7b,c show little difference in the feature distributions, which indicates that the traditional transfer learning strategy has a limited effect on the improvement of the baseline FCN model.

By analyzing the load identification results with the t-SNE feature maps, the supervised scene adaptation model could accomplish the transfer better even under the completely different conditions of regional structure. Under the condition of very few samples, the supervised scene adaptation model identification was much better than the baseline FCN model and TL-FCN model.

**(3) Transfer ability of model under different impact energy scenarios**

This subsection further investigated the impact of impacts in different energy level conditions on the model for load identification. In order to make the load identification capability of the model cover various energy impacts, traditional deep learning methods could only simulate or collect impact response signals of small and large energy level impacts in large quantities to improve the generalization of the model to different energy impacts. And since multiple large-energy impacts might cause damage to the aircraft structure, it would mean that large-energy impact experiments relying on actual platforms cannot be repeated in large numbers.

First, the transfer capability of the model under different energy level impacts was validated. Combined with the problems faced in engineering applications, the sample set under small-energy impact events was selected in this section as the baseline set to train the model, and then a small amount of impact response signals generated by large-energy impact events were collected to calibrate the model. The small-energy impact dataset A1S from Area1 and the large-energy impact dataset A1B were selected here. A1S was used as the baseline set to complete the basic training of the model, while A1B was divided into a calibration set and test set according to a certain ratio, and the calibration set was used to calibrate the model for new scenarios, while the test set was used to test the prediction of the model under the large-energy impact.

**Figure 8:**
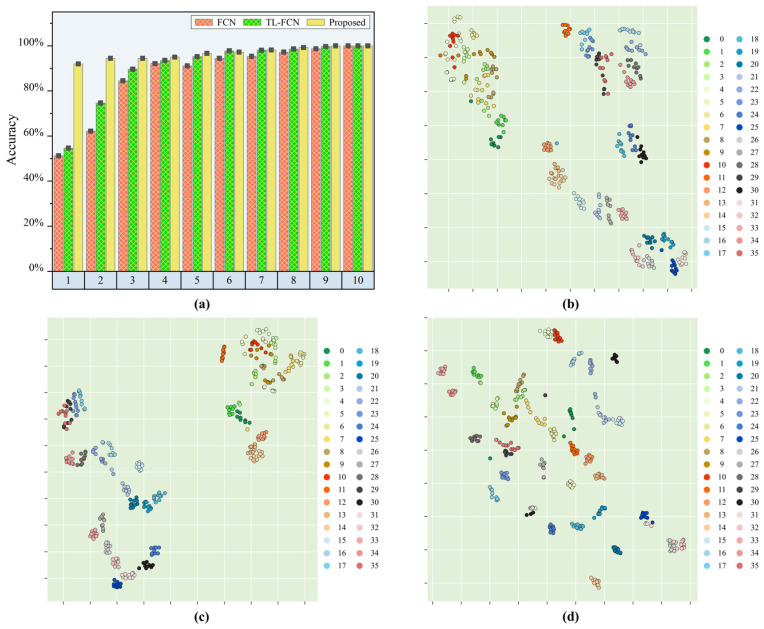
Load position identification effect of each model under different energy level impact conditions. (**a**) Identification accuracy of each model; (**b**) feature visualization of the baseline FCN model; (**c**) feature visualization of TL-FCN model; (**d**) feature visualization of supervised scene adaptation model.

After a series of experimental validation, the results are shown in Figure 8 and Table 4. The load identification accuracy of the supervised scene adaptation model reached 91.98% when the number of calibration samples in a single sub-area was 1, which was 40.75% higher than the load identification accuracy of the baseline FCN model. In contrast, the identification accuracy of the traditional transfer learning-based FCN model only improved by 3.4%, which was far from the accuracy standard required for practical engineering applications. The proposed method achieved 94.44% load recognition accuracy when the calibration sample was 2, while the identification accuracies of the baseline FCN model and the traditional deep learning-based FCN model were only 62.15% and 74.65%, respectively. The baseline FCN model and the traditional transfer learning-based FCN model showed better load identification ability only when the calibration sample size reached 4 or more. Meanwhile, the supervised scene adaptation model had about 2% prediction performance improvement compared to the baseline FCN model.

In order to obtain visualization of the transfer learning strategy and the domain adaptation strategy, the output of the fully connected layer was transformed to a two-dimensional space using t-SNE and then a scatter plot was drawn. Here, a calibration dataset with one sample for a single sub-area and a test set with ten samples for a single sub-area are selected. Figure 8b–d present the feature distribution plots of the FCN model, TL-FCN model and supervised scene adaptive model for the prediction of the test set. They had similar feature distributions, but the supervised scene adaptation model had a larger and more dispersed interval distance between each category compared to the baseline FCN model and TL-FCN model, which explained from the side why the supervised scene adaptation model still had better load identification with very few samples.

The above identification results with t-SNE feature analysis demonstrated that the supervised scene adaptation model could effectively improve the generalization to different energy impacts. Compared with the baseline FCN model and TL-FCN model, the accuracy of load identification in the case of few samples with very few samples was greatly improved.

**(4) Transfer ability of model with completely different scenarios of impact energy level and area/structure**

Past studies divided and discussed the situations encountered in practical scenarios, and did not sufficiently analyze the situation where multiple complex conditions might be encountered in practical application scenarios. Therefore, this subsection further validates the effectiveness of the proposed model for load identification in mixed scenarios with different impact energy levels and different area structures, and further demonstrates whether the model could achieve high performance transfer for any energy impact with any structure area.

Combined with some problems faced by the practical application of engineering, sufficient small-energy impact experiments were conducted in the benchmark area, and then small amounts of large-energy impact samples were collected in the target area to complete the validation of the model’s generalization ability under complex conditions. Here, the small-energy impact dataset A1S from Area1 was selected as the benchmark set, and the large-energy impact dataset A3B from Area3 was divided into a calibration set and test set in a certain proportion to complete the calibration and performance testing of the model for new scenarios.

**Figure 9:**
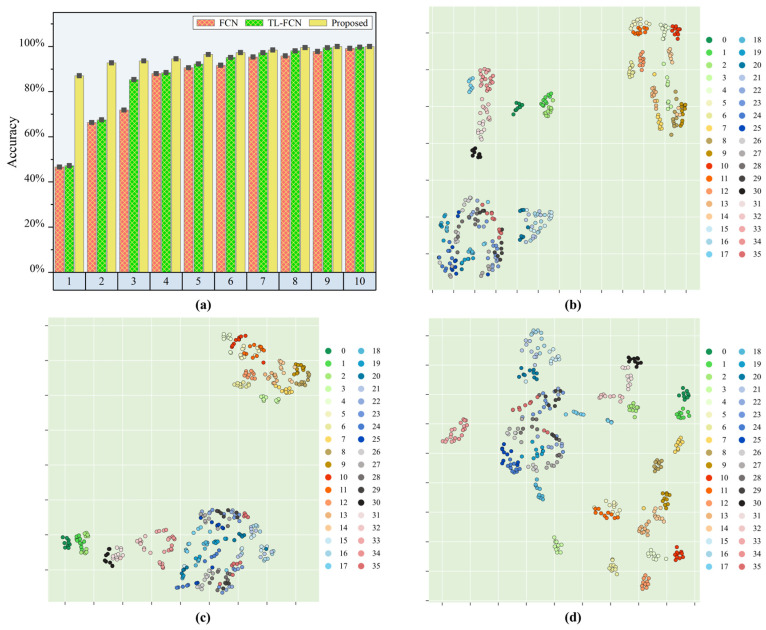
Load position identification effect of each model under different impact energy levels and different area structure conditions. (**a**) Identification accuracy of each model; (**b**) feature visualization of the baseline FCN model; (**c**) feature visualization of the TL-FCN model; (**d**) feature visualization of the supervised scene adaptation model.

The load identification results of the experimentally validated model are shown in Figure 9 and Table 5. The load identification accuracy of the supervised scene adaptation model was nearly twice as high as that of the baseline FCN model and the traditional transfer learning-based FCN model, reaching 87.04% for one single sub-area calibration sample. In contrast, the identification accuracy of both the baseline FCN model and the traditional transfer learning-based FCN model was below 50%. When the calibration samples totaled 2, the load identification accuracy of the proposed model reached 92.71%, which was improved by 26.39% compared to the benchmark FCN model. In contrast, the FCN model based on traditional transfer learning improved by only 1.18% compared to the original model. The supervised scene adaptation model achieved identification accuracy of 93.60% when the calibration samples totaled 3, which was improved by 21.77% compared to the original model. The identification accuracies of the baseline FCN model, the traditional transfer learning-based FCN model, and the supervised scene adaptation model all reached more than 90% when the calibration samples for each subregion were larger than 4. And as the sample size continued to increase, the performance improvement that the supervised scene adaptation model could bring gradually reached the limit.

To explain the results presented in the experiments, t-SNE was further used to convert the fully connected layer high-dimensional output vectors of the baseline FCN model, the pre-trained TL-FCN model, and the supervised scene adaptation model into two-dimensional vectors. The feature distributions of the FCN model, the TL-FCN and the supervised scene adaptation model are shown in Figure 9b–d. The distributions of the extracted features of the three models were similar. Compared with the baseline FCN model, the distribution interval density of the TL-FCN extracted features did not change much, so the TL-FCN model was poorly boosted in the case of very few samples. The supervised scene adaptation model extracted more discrete feature distribution intervals for different sub-areas of the impact response signal. Therefore, the supervised scene adaptive model could accomplish high-accuracy identification of loads with very few samples for training.

This subsection further validated the load identification effect of the proposed model in mixed scenarios with different impact energy levels and different area structures, and analyzed the load identification results with t-SNE feature maps. The results demonstrated that the supervised scene adaptation model could be effectively applied to practical scenarios where multiple complex conditions coexist. Even in the scenarios with different impact energy levels and different regional structures, the supervised adaptation model still performed well with very few samples and could be well suited for practical engineering applications.

### 3.3. Energy Identification of Impact Load

In practical engineering application scenarios, predicting the energy of external impacts was critical for assessing the extent of damage that might be caused to the structure internally by the impact. This section further validated the ability of the supervised scene adaptation model to perform impact load energy identification and discussed the transfer ability of the model to perform impact energy identification in similar areas/structures versus different areas/structures. Here, the supervised scene adaptive model was compared with the baseline FCN model and the transfer learning-based TL-FCN model to validate whether the proposed model had any improvement in generalization ability compared with the traditional deep learning model with the introduction of the transfer learning strategy. This subsection first discusses the transfer effect of the model for load energy identification in the similar area structure scenario, and verifies whether the model could accomplish more effective energy level evaluation in similar structures. Considering that the practical application scenarios involved the transfer of the model under different structural conditions, this section further discusses the transfer effect of the model for load energy level identification under different area structure scenarios.

**(1) Transfer ability of model in similar area/structure scenarios**

The transfer performance of the model was verified under similar area structure scenarios. Here, four datasets from Area1 and Area2 were selected for mixing and partitioning. A1S and A1B from Area1 were mixed and labeled to form the A1 hybrid dataset, and dataset A1 was used as the baseline set. Further, A2S and A2B from Area2 were mixed to obtain the A2 mixed dataset. Then, the A2 mixed dataset was divided according to a certain ratio to obtain the calibration set and test set to complete the calibration and testing of the model.

**Figure 10:**
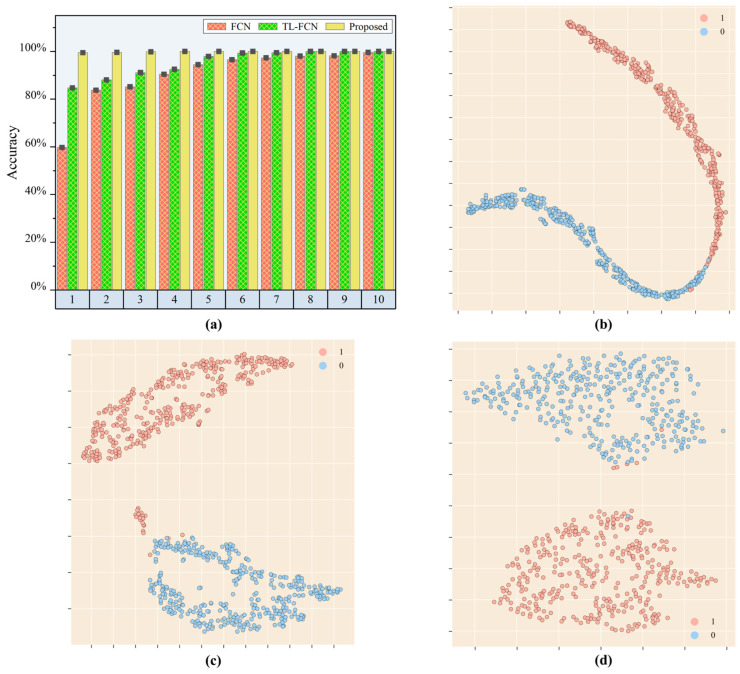
Impact energy identification effect of each model in similar area/structure scenes. (**a**) Identification accuracy of each model; (**b**) feature visualization of the baseline FCN model; (**c**) feature visualization of TL-FCN model; (**d**) feature visualization of supervised scene adaptation model.

After a series of experimental validation, the result can be obtained as illustrated in Figure 10 with Table 6. Figure 10a displays the prediction results of the transfer model under similar area structure scenes. The supervised scene adaptation model reached more than 99% accurate identification rate when there were one to three samples for each impact energy level. In particular, the supervised scene adaptation model improved by 39.72% compared to the baseline FCN model when there was one sample per impact energy level, while the traditional transfer learning-based TL-FCN model improved by only 24.99%. With four samples per impact energy level, the supervised scene adaptation model accomplished 100% accurate identification rate. The baseline FCN model achieved only 90.42% accuracy. The accuracy of the baseline FCN model only reached over 95% when the calibration samples totaled six, and 99% when the calibration samples totaled ten. Meanwhile, the identification accuracy of the TL-FCN model based on traditional transfer learning was only about 3% higher than that of the baseline FCN model, which indicated that the optimization effect of traditional transfer learning was relatively limited.

In order to explain the results presented by the experiments, the high-dimensional vectors of the fully connected layers of each model were further downscaled into two-dimensional vectors using t-SNE. Here, the distribution of features presented by each model after feature extraction for test sets of 20 samples per energy level was analyzed when the calibration data set included one sample per energy level. From Figure 10b–d, it could be observed that the feature distributions extracted by the baseline FCN model for the impact response signals of different energy levels overlapped severely. TL-FCN was able to separate the features of different impact energy levels to some extent, but there was still some crossover in the feature distributions extracted by TL-FCN. The separation effect of the supervised scene adaptation model was better compared to TL-FCN, which fully achieved the dispersion isolation of features with different energy levels. In this validation experiment, the enhancement effect of TL-FCN on the energy level identification of loads was better than the location identification of loads due to the fact that the energy level identification task involves more distinct features relative to the location identification of loads, and the distribution of samples collected from different regional structures has some similarity. The superior identification effect of the supervised scene adaptation model over TL-FCN was due to its alignment idea, where data from different scenes were projected into a common feature space and aligned.

This subsection further validates the proposed model for load energy identification under similar regional structure scenarios, and analyzes the load identification results with t-SNE feature maps. The results indicate that the transfer effect of the proposed model for load energy identification under similar area structure was better, and the performance was still superior in the case of very few samples, which could better fit the practical engineering applications.

**(2) Transfer ability of model in completely different scenarios of area/structure**

The transfer capability of the model for load energy identification under different conditions of the area structure was validated. Combined with the practical engineering application scenarios, the small-energy impact dataset A1S from Area1 was mixed with the large-energy impact dataset A1B to obtain dataset A1. Then, the small-energy impact dataset A3S from Area3 was mixed with the large-energy impact dataset A3B to obtain dataset A3, and then the calibration set and test set were divided according to certain ratio. The calibration set and test set were then divided according to a certain ratio.

**Figure 11:**
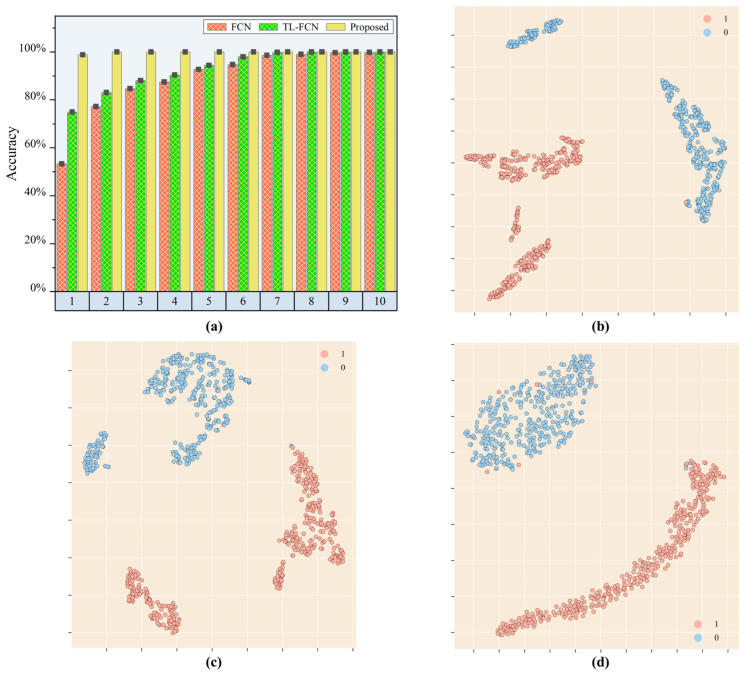
Impact energy identification effect of each model in different scenarios of area/structure. (**a**) Identification accuracy of each model; (**b**) feature visualization of the baseline FCN model; (**c**) feature visualization of TL-FCN model; (**d**) feature visualization of supervised scene adaptation model.

Figure 11a and Table 7 show the transfer performance of each model for load energy identification under different region structures. The supervised scene adaptation model attained 98.85% accuracy with one calibration sample and 100% accuracy with two calibration samples. In contrast, the baseline FCN model only achieves 90.14% with five calibration samples. The improvement effect of the TL-FCN model based on traditional transfer learning was relatively limited, with only 21.59% improvement with one calibration sample and 5.84% improvement with two calibration samples, and the improvement effect diminished when the number of calibration samples was increased gradually. Through the above results, the supervised adaptive model was better than the traditional transfer learning model in performing the load energy identification task, and had better transfer effects for different regional structures, which could meet the practical engineering application requirements.

Figure 11a and Table 7 show the transfer performance of each model for load energy identification under different area structures. The supervised scene adaptation model attained 98.85% accuracy with one calibration sample and 100% accuracy with two calibration samples. In contrast, the baseline FCN model only achieved 90.14% with five calibration samples. The improvement effect of the TL-FCN model based on traditional transfer learning was relatively limited, with only 21.59% improvement with one calibration sample and 5.84% improvement with two calibration samples, and the improvement effect diminished when the number of calibration samples was increased gradually. Through the above results, the supervised adaptive model was better than the traditional transfer learning model in implementing the load energy identification task, and had better transfer effects for different regional structures, which could meet the practical engineering application requirements.

To obtain visualization of the transfer learning strategy and the adaptive strategy, the output features of the fully connected layer were dimensioned down to two-dimensional features using t-SNE and then converted into scatter plots. Here, the calibration dataset of one sample for each energy level and the test set of twenty samples for each energy level were selected. Figure 11b–d represent the distribution of features predicted by the FCN model, TL-FCN model and supervised scene adaptation model for the test set. The features extracted by the FCN model, TL-FCN model and supervised scene adaptation model had similar distribution. However, the supervised scenario adaptation model resulted in greater distance between the impact response signals of different energy levels and tighter clustering of impact response signals of the same energy level compared with the baseline FCN model and TL-FCN model. The feature distribution diagram effectively illustrates why the supervised scene adaptive model was better in load identification even with very few samples.

The above identification results and the t-SNE feature analysis demonstrate that the supervised scene adaptation strategy could effectively improve the generalization of the model to perform the impact load energy identification task, even under the condition of completely different area structures, and still accomplish the accurate prediction of the load energy level with very few samples.

### 3.4. Transfer Learning Under Different Structures

**Figure 12:**
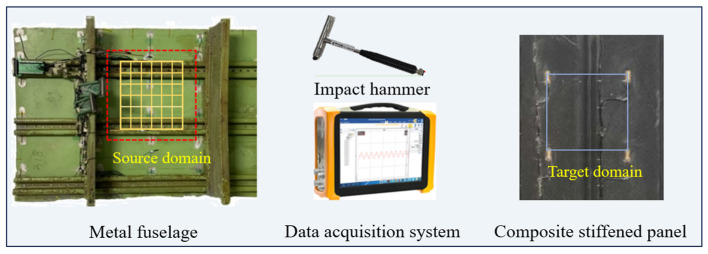
Transfer learning verification experiment between different structures.

As shown in Figure 12, based on the above research, further research has been carried out on the transfer ability of the proposed method on different structures. The source domain is still set on Area1 of the metal fuselage in Figure 5, and the dataset is A1B. The target domain is a composite-reinforced panel, which has a different material and stiffener with the metal fuselage, but the monitoring dimensions are the same as Area1 in the metal fuselage. The dataset collection is the same as A3B of the metal fuselage, and the dataset is divided into calibration and testing sets according to a certain proportion. The transfer ability from the metal fuselage to the composite-reinforced plate is verified.

Table 8 shows the accuracy of impact location identification on the composite-reinforced panel. When there is one calibration sample for each subregion, the recognition accuracy of the proposed method reaches 83.37%, which is much higher than FCN and TL-FCN. The TL-FCN model based on traditional transfer learning does not show significant changes in prediction accuracy compared to the benchmark FCN model when the calibration sample is 1. When the number of calibration samples in each subregion is increased to 2, the accuracy of impact location identification in the proposed method approaches 90%. The proposed model performs well in terms of few samples in different structural scenarios and has good engineering applicability.

Table 9 shows the accuracy of impact energy identification on the composite-reinforced panel. The accuracy of the method proposed in this article reaches 94.81% when the calibration sample is 1, and 95.04% when the calibration sample is 2. In contrast, the benchmark FCN model and TL-FCN only achieved 50.96% and 71.67%, respectively, with a single calibration sample, and only reached 90.14% with five calibration samples. The method proposed in this article has better transfer performance in performing energy recognition tasks with few samples, and can meet the needs of practical engineering applications.

## 4. Conclusions

In this paper, a few-sample impact load identification method based on a supervised scene adaptation model is presented. Supervised scene adaptation enables mapping the feature and label distributions of structures in different areas to the same space, providing a novel solution to the problem. For large and complex structures, the model can be applied to other similar structural areas or different structural areas by using samples from the baseline area for training, and then using a very small number of calibration samples from the migrated area to participate in the calibration. Also, t-SNE is introduced in this paper to visualize and analyze the features extracted from the model, and further visualize and verify the transfer characteristics of the proposed model.

The analysis of the experiment results led to the following conclusions:

(1) Under similar area structure conditions, the supervised scene adaptation model achieves 97.22% accuracy in impact load location identification and 99.44% accuracy in impact load energy identification after one-sample calibration in each sub-area. Under different structural area conditions, the impact load location identification accuracy is 87.65%, and the impact load energy identification accuracy is 98.85%.

(2) Under different impact energy levels, the supervised scene adaptation model reaches 91.98% accuracy in impact load location identification after one-sample calibration in each sub-area. The impact load location identification accuracy achieves 87.04% under different impact energy levels and different structural areas.

(3) t-SNE is introduced to visualize and analyze the features extracted by the model, and validate whether the proposed model achieves aggregation of similar features and dispersion of different classes of features in the process of feature extraction and common mapping.

The training and testing data for the impact load identification method in this article were collected in a laboratory environment, which has good consistency. However, in actual operating environments, the training data collected on the ground and the actual flight testing data environment are different, and the influence of time-varying environments such as temperature and vibration has an impact on the impact stress wave. How to correct these influencing factors so that the method can also be prepared for identification in time-varying environments is a potential direction that needs to be considered in the future.

## Figures and Tables

**Table 1:** Dataset of impact experiments.

Impact Location	Dataset Name	Energy Level (N)	Number of Impacts per Position	Dataset Size
Area1	A1S	0 < *n* < 200	10	360
Area1	A1B	*n* > 200	10	360
Area2	A2S	0 < *n* < 200	5	180
Area2	A2B	*n* > 200	5	180
Area3	A3S	0 < *n* < 200	5	180
Area3	A3B	*n* > 200	5	180

**Table 2:** Accuracy of load location identification for each model in similar area/structure scenarios.

Model	Number of Calibration Samples									
	1	2	3	4	5	6	7	8	9	10
FCN	55.25%	76.74%	79.76%	93.06%	97.22%	98.64%	99.44%	99.78%	100%	100%
TL-FCN	55.83%	77.43%	88.89%	93.98%	98.54%	99.21%	100%	100%	100%	100%
Proposed	97.22%	99.30%	99.60%	100%	99.44%	99.30%	100%	100%	100%	100%

**Table 3:** Accuracy of load location identification for each model in different area/structure scenarios.

Model	Number of Calibration Samples									
	1	2	3	4	5	6	7	8	9	10
FCN	42.28%	71.53%	86.11%	86.57%	92.78%	95.83%	96.30%	96.58%	99.17%	100%
TL-FCN	45.68%	74.17%	88.89%	90.28%	95.56%	96.56%	98.89%	97.78%	99.72%	100%
Proposed	87.65%	93.75%	94.44%	96.76%	96.66%	97.22%	99.07%	98.61%	100%	100%

**Table 4:** Accuracy of load location identification for each model under different energy level impact conditions.

Model	Number of Calibration Samples									
	1	2	3	4	5	6	7	8	9	10
FCN	51.23%	62.15%	84.52%	92.13%	91.11%	94.44%	95.37%	97.22%	98.75%	100%
TL-FCN	54.63%	74.65%	89.68%	93.52%	95.22%	97.78%	98.07%	98.61%	99.72%	100%
Proposed	91.98%	94.44%	94.44%	94.98%	96.66%	97.22%	98.15%	99.22%	100%	100%

**Table 5:** Accuracy of load location identification for each model under different impact energy levels and different area structure conditions.

Model	Number of Calibration Samples									
	1	2	3	4	5	6	7	8	9	10
FCN	46.60%	66.32%	71.83%	87.96%	90.56%	91.67%	95.37%	95.83%	97.78%	99.17%
TL-FCN	47.22%	67.50%	85.32%	88.43%	92.22%	95.14%	97.22%	98.06%	99.44%	99.72%
Proposed	87.04%	92.71%	93.60%	94.54%	96.44%	97.31%	98.45%	99.53%	100%	100%

**Table 6:** Accuracy of impact energy identification for each model in similar area structure scenarios.

Model	Number of Calibration Samples									
	1	2	3	4	5	6	7	8	9	10
FCN	59.72%	83.75%	85.14%	90.42%	94.44%	96.53%	97.31%	98.01%	98.15%	99.57%
TL-FCN	84.72%	88.06%	91.11%	92.50%	97.92%	99.32%	99.44%	100%	100%	100%
Proposed	99.44%	99.58%	99.85%	100%	100%	100%	100%	100%	100%	100%

**Table 7:** Accuracy of impact energy identification for each model under different scenarios of area structure.

Model	Number of Calibration Samples									
	1	2	3	4	5	6	7	8	9	10
FCN	53.33%	77.22%	84.72%	87.50%	92.73%	94.72%	98.61%	99.03%	99.72%	99.86%
TL-FCN	74.92%	83.06%	88.06%	90.42%	94.44%	97.98%	99.86%	100%	100%	100%
Proposed	98.85%	100%	100%	100%	100%	100%	100%	100%	100%	100%

**Table 8:** Accuracy of impact location identification under different structures.

Model	Number of Calibration Samples									
	1	2	3	4	5	6	7	8	9	10
FCN	40.67%	67.95%	83.29%	83.22%	90.36%	92.78%	95.00%	94.64%	98.55%	99.61%
TL-FCN	42.90%	70.96%	84.65%	87.30%	91.69%	93.99%	95.86%	96.31%	97.41%	99.50%
Proposed	83.37%	89.09%	90.86%	92.75%	93.75%	93.91%	97.46%	95.93%	98.56%	100%

**Table 9:** Accuracy of impact energy identification under different structures.

Model	Number of Calibration Samples									
	1	2	3	4	5	6	7	8	9	10
FCN	50.96%	73.48%	81.78%	84.19%	90.35%	91.71%	97.12%	96.46%	98.96%	99.22%
TL-FCN	71.67%	78.96%	85.13%	86.94%	91.78%	94.66%	98.42%	98.91%	100%	100%
Proposed	94.81%	95.04%	96.19%	95.83%	96.98%	97.92%	98.27%	100%	100%	100%

## Data Availability

Data will be made available on request.
